# A Time-Saving Strategy to Generate Double Maternal Mutants by an Oocyte-Specific Conditional Knockout System in Zebrafish

**DOI:** 10.3390/biology10080777

**Published:** 2021-08-16

**Authors:** Chong Zhang, Jiaguang Li, Imran Tarique, Yizhuang Zhang, Tong Lu, Jiasheng Wang, Aijun Chen, Fenfen Wen, Zhuoyu Zhang, Yanjun Zhang, Ming Shao

**Affiliations:** 1 Shandong Provincial Key Laboratory of Animal Cell and Developmental Biology and Key Laboratory for Experimental Teratology of the Ministry of Education, School of Life Sciences, Shandong University, Qingdao 266237, China; zhangchong@mail.sdu.edu.cn (C.Z.); samoo_imran88@hotmail.com (I.T.); zhangyizhuang@mail.sdu.edu.cn (Y.Z.); 202020327@mail.sdu.edu.cn (T.L.); wjs2423xx@mail.sdu.edu.cn (J.W.); 202012514@mail.sdu.edu.cn (A.C.); 202012576@mail.sdu.edu.cn (F.W.); zhangyj@sdu.edu.cn (Y.Z.); 2 Taishan College, Shandong University, Qingdao 266237, China; 201800140144@mail.sdu.edu.cn (J.L.); 201800140004@mail.sdu.edu.cn (Z.Z.)

**Keywords:** maternal factors, oocyte, CRISPR/Cas9, double mutant, early development, zebrafish, conditional knockout

## Abstract

**Simple Summary:**

Maternally supplied mRNAs and proteins, termed maternal factors, are produced by over 14,000 coding genes in zebrafish. They play exclusive roles in controlling the formation of oocytes and the development of early embryos. These maternal factors can also compensate for the loss of function of its corresponding zygotic gene products. Thus, eliminating both maternal and zygotic gene products is essential to elucidate the functions of more than half of zebrafish genes. However, it is always challenging to inactivate maternal factors, because traditional genetic methods are either technically demanding or time-consuming. Our recent work established a rapid conditional knockout method to generate maternal or maternal and zygotic mutants in one fish generation. Here, we further test the feasibility of this approach to knock out two maternal genes with functional redundancy simultaneously. As a proof of principle, we successfully generated double maternal mutant embryos for *dvl2* and *dvl3a* genes in three months for the first time. The cell movement defects in mutant embryos obtained by this approach mimic the genuine mutant embryos generated after fifteen months of time-consuming screening following the previously reported mosaic strategy. Therefore, this method has the potential to speed up the functional study of paralogous maternal genes.

**Abstract:**

Maternal products are those mRNAs and proteins deposited during oogenesis, which play critical roles in controlling oocyte formation, fertilization, and early embryonic development. However, loss-of-function studies for these maternal factors are still lacking, mainly because of the prolonged period of transgenerational screening and technical barriers that prevent the generation of maternal (M) and maternal and zygotic (MZ) mutant embryos. By the transgenic expression of multiple sgRNAs targeting a single gene of interest in the background of a transgenic line Tg(*zpc*:*zcas9*) with oocyte-specific *cas9* expression, we have successfully obtained maternal or maternal–zygotic mutant for single genes in F1 embryos. In this work, we tandemly connected a maternal GFP marker and eight sgRNA expression units to target *dvl2* and *dvl3a* simultaneously and introduced this construct to the genome of Tg(*zpc*:*zcas9*) by meganuclease I-*Sce* I. As expected, we confirmed the existence of M*dvl2*;M*dvl3a* embryos with strong defective convergence and extension movement during gastrulation among outcrossed GFP positive F1 offspring. The MZ*dvl2*;MZ*dvl3a* embryos were also obtained by crossing the mutant carrying mosaic F0 female with *dvl2*^+/−^;*dvl3a*^−/−^ male fish. This proof-of-principle thus highlights the potential of this conditional knockout strategy to circumvent the current difficulty in the study of genes with multiple functionally redundant paralogs.

## 1. Introduction

Maternal contribution is indispensable to drive early embryonic development, which is essentially “My mother made me do it” [1]. During oogenesis in zebrafish, meiosis temporally halts in a prolonged diplotene stage, when mRNAs and proteins are synthesized and stored for oocyte maturation, fertilization, and early development [2,3,4,5,6,7,8]. The gene products accumulated at this stage are termed maternal factors, which are generated by over half of the coding genes in zebrafish [9]. Maternal factors play a crucial role in controlling early embryonic development before the zygotic genome activation and can act as a buffering mechanism to compensate for the loss of their corresponding zygotic gene products [10,11,12]. Thus, the generation of maternal–zygotic (MZ) mutants is essential to thoroughly elucidate the roles of genes with maternal expression in early development.

Because primary oocytes have the same genotype as somatic cells, viable and fertile homozygous mutant female fish are the prerequisite to obtain maternal (M) and MZ mutants. In zebrafish, the traditional genetic method takes three generations to obtain adult homozygous mutant female fish for a single maternal gene. This time will almost double when screening a mutant female with two homozygous mutant genes from the initial Cas9 ribonucleoprotein (RNP) injection step [10]. However, if homozygous mutation of zygotic gene products causes death or sterility, the removal of maternal factors requires special methods with high technical barriers. These strategies include germ-line replacement [13,14,15], oocyte microinjection in situ [16], the mosaic approach [10], and the BAC conditional rescue (BACK approach) [17]. As such, there is an urgent need to establish a simple and time-saving genetic method for generating maternal mutants.

In our recent work, we efficiently generated *nanog* and *ctnnb2* maternal mutants in just one fish generation by introducing sgRNA expression cassettes in the background of Tg(*zpc*:*zcas9*), which expresses zebrafish codon-optimized Cas9 under the control of the zona pellucida gene *zp3b* [18,19]. Compared to previous methods, this novel oocyte-specific conditional knockout (CKO) strategy presents several advantages. First, it is much more time-saving, as the time to get M or MZ mutants can be reduced to about three months. Second, this method is also efficient in generating maternal mutants of single genes; after expressing three sgRNAs targeting a gene of interest, more than 25% of GFP-positive embryos are expected to be the maternal mutant [18]. Notedly, this transgenic expression of multiple Cas9 RNPs in the oocyte can generate large deletions on the genes of interest with high efficiency and thus can theoretically prevent potential genetic compensation [18]. However, the effect of this strategy to generate double maternal mutants has not been tested.

In many cases, paralogs can work together and mutually compensate for the loss of their functions [20]. Hence, to study functionally related genes, the knockout of two or even more genes at a time is demanded. Take *dishevelled* (*dvl*) genes as an example; *dvl2* and *dvl3a* are the main paralogs highly expressed during the early development of zebrafish. They are functionally redundant in controlling Wnt/PCP and zygotic Wnt/β-catenin activation [10,21]. Loss of *dvl3a* produced no apparent phenotypes, and similarly, *dvl2* zygotic mutants showed relatively normal development. Only eliminating the maternal and zygotic gene products of both genes can cause the most severe developmental defects, including extreme anteriorization and defective convergence extension movements [10]. Double zygotic mutants of these genes are lethal, so it is impossible to generate double maternal mutant embryos via traditional methods. Using a mosaic approach in our previous work, double maternal mutants of these two genes were obtained only after more than one year (five generations) of laborious efforts [10]. So, it is fascinating to test whether double maternal mutants could be generated through the new oocyte CKO approach in only one generation.

This work aimed to test the feasibility of using the oocyte-specific CKO strategy to generate double maternal mutants in zebrafish. By employing the recently established oocyte-specific CKO strategy, we successfully obtained double mutant embryos in one fish generation by the expression of four *dvl2* sgRNAs and four *dvl3a* sgRNAs in Tg(*zpc*:*zcas9*) fish. Compared to the traditional way of obtaining double maternal mutants in zebrafish, this strategy overcomes the obstacles of zygotic lethal phenotypes and is extremely time-saving. This proof-of-principle thus demonstrates the potential of this novel CKO strategy in functional studies of paralogous maternal genes in zebrafish.

## 2. Materials and Methods

### 2.1. Zebrafish Maintenance

Wild type AB and Tg(*zpc*:*zcas9*) were maintained in standard conditions. The Tg(*zpc*:*zcas9*) was previously described [18] and is available in China Zebrafish Resource Center (CZRC). Embryos were raised at 28.5 °C. Animal experiments were designed and executed under guidelines of the Animal Research: Reporting of In Vivo Experiments (ARRIVE), and were approved by the Ethics Committee for Animal Research of Life Science of Shandong University (permit number SYDWLL-2018-05).

### 2.2. sgRNA Design and Efficiency Test

sgRNAs targeting *dvl2* or *dvl3a* were designed using the online algorithm CRISPRScan (https://www.crisprscan.org/, accessed on 8 January 2004) and listed in Table S1. The sgRNA DNA templates were amplified using Fill-in PCR. The universal primer sequence was 5′-AAAAGCACCGACTCGGTGCCACTTTTTCAAGTTGATAACGGACTAGCCTTATTTTAACTTGCTATTTCTAGCTCTAAAAC-3′. After purification of sgRNA DNA template, sgRNA was in vitro transcribed and cleaned up by phenol–chloroform extraction, followed by precipitation by isopropanol. To test the sgRNA efficiency, the mixture (150 pg each) of sgRNA and zebrafish codon-optimized *cas9* (*zcas9*) mRNA [22] was injected into 1-cell stage wild type embryos. At 10 h post fertilization (hpf), ten injected embryos were collected and lysed as described [9]. Then, the lysate was used to amplify the fragment containing the sgRNA site, which was subsequently subjected to Sanger sequencing. The quantification of sgRNA efficiency was performed through analyzing the sequencing chromatogram files using the online algorithms 

### 2.3. Plasmids Construction

Complementary primer pairs containing the protospacer sequences of selected sgRNA were annealed and separately cloned into the *Bsm*BI sites of pU6:sgRNA1-4 (pU6a:sgRNA#1, pU6a:sgRNA#2, pU6b:sgRNA#3, and pU6c:sgRNA#4) to generate sgRNA expression cassettes driven by U6a, U6b or U6c promoter. The four *dvl2* sgRNA expression modules in pU6:sgRNA1-4 were tandemly ligated by Golden Gate ligation to create a pGGDestISceIEG-4sgRNA(*dvl2*) vector [23]. Following the same procedure, we also generated a pGGDestISceIEG-4sgRNA(*dvl3a*) vector containing a stretch of expression modules of the four *dvl3a* sgRNAs. Finally, the four *dvl3a* sgRNA expressing cassettes were amplified from pGGDestISceIEG-4sgRNA(*dvl3a*) and ligated into the *Asp*718I site of pGGDestISceIEG-4sgRNA(*dvl2*) vector by Gibson assembly [24]. Therefore, after these cloning steps, we obtained a pGGDestISceIEG-8sgRNA(*dvl2*;*dvl3a*) transgenic vector that could drive the expression of the four sgRNAs targeting *dvl2* and four sgRNAs against *dvl3a*. This 8-sgRNA expression vector contains two I-*Sce* I sites for transgenesis and a maternal GFP marker for maternal mutant selection. The primers were listed in Table S1.

### 2.4. Transgenesis of sgRNA Expression Vector via I-Sce I

A mixture of I-*Sce* I (1 u/μL, NEB), plasmids (7.5 ng/μL), and CutSmart buffer (0.5×, NEB) was injected into 1-cell stage embryos from Tg(*zpc*:*zcas9*) or wild type background [25]. To ensure the activity of I-*Sce* I, part of the mixture was incubated at 37 °C for 30 min and then analyzed by gel electrophoresis. At 4 dpf, embryos displaying strong and widespread GFP signals were raised to adulthood.

### 2.5. Quality Control before Screening Maternal Mutants

GFP-negative embryos from the mosaic founder female were injected with 100 pg sgRNA against *bmp2b* [19]. Only when they were efficiently dorsalized could the phenotype of GFP-positive counterparts be further analyzed.

### 2.6. Ploidy Analysis by Quantitative PCR

A wild-type and an embryo with the M*dvl2*;M*dvl3a* mutant phenotype were lysed at 30 hpf, and their genomic DNAs were extracted. Quantitative PCR was performed using 2× M5 HiPer SYBR Premix (MF787-01, Mei5) on a Q1000 Real-Time PCR System (LongGene). The primers for amplifying *dvl2*, *dvl3a*, and *dmd* genomic regions are listed in Table S1. The 2^−ΔΔCt^ method was employed to estimate ploidy at these loci. The average copy number of these amplicons in wild-type embryos was normalized as two. The *dmd* gene is located on a different chromosome (chr1) from *dvl2* (chr7) and *dvl3a* (chr2), and has no potential off-target sites for the eight sgRNAs used in this study. So, this gene should be diploid in both wild-type and M*dvl2*;M*dvl3a* embryos, and thus the amplicon from this gene is suitable to serve as a diploid reference or loading control in qPCR experiments.

### 2.7. Total RNA Extraction and RT-PCR

Two-cell-stage embryos were lysed individually in 200 μL Trizol. Total RNA was purified using phenol-chloroform and precipitated using isopropanol. cDNAs were synthesized using the cDNA First-Strand Synthesis SuperMix (Transgene, AT301) and then subjected to CDS amplification using the primers listed in Table S1. The PCR products were analyzed by gel electrophoresis followed by Sanger sequencing.

## 3. Results

### 3.1. Experimental Design to Generate Double Maternal Mutants

The core idea of the oocyte CKO is to introduce transgenic sgRNA expression cassettes in the background of the Tg(*zpc*:*zcas9*) fish. As the *zpc* promoter can drive the expression of *zcas9* specifically in the early stage oocyte [19], the broad expression of sgRNAs thus can allow maternal knockout of genes of interest. For this purpose, zebrafish *dvl2* and *dvl3a* were selected as targets, because they display both redundant and distinct functions in embryonic axis formation and morphogenetic movements in vertebrate embryos [21]. As the generation of double maternal mutants of *dvl2* and *dvl3a* is extraordinarily time-consuming and labor-intensive, we wondered if double maternal mutants could be generated through this conditional approach in only one generation.

**Figure 1:**
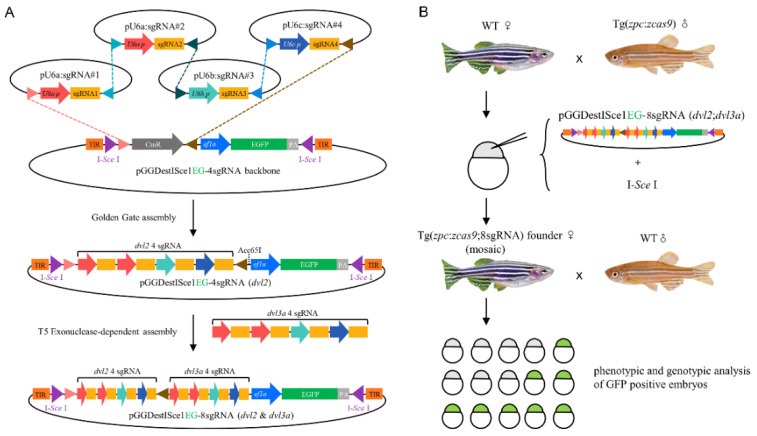
Workflow to generate double maternal mutants for *dvl2* and *dvl3a* genes. (**A**) Two cloning steps to generate transgenic vectors containing eight sgRNA expression cassettes. By Golden Gate ligation, four sgRNA expression cassettes are cloned tandemly into pGGDestISceIEG-4sgRNA backbone, generating pGGDestISceIEG-4sgRNA (*dvl2*) and pGGDestISceIEG-4sgRNA (*dvl3a*). The four sgRNAs targeting *dvl3a* were then amplified from the pGGDestISceIEG-4sgRNA (*dvl3a*) plasmid and inserted into the *Acc*65I site in pGGDestISceIEG-4sgRNA (*dvl2*) vector via T5 exonuclease-dependent assembly (TEDA). (**B**) The resultant pGGDestISceIEG-8sgRNA (*dvl2*;*dvl3a*) was coinjected with I-*Sce* I into embryos spawned by a wild-type female and a Tg(*zpc*:*zcas9*) homozygous male. The mosaic female with germ-line transmission was outcrossed to produce GFP-positive embryos, which were then genotyped and phenotyped for double maternal mutations.

As multiple transgenic Cas9 RNPs targeting a single gene of interest can increase the editing efficiency [26], we designed a transgenic vector containing four *dvl2* sgRNAs and four *dvl3a* sgRNAs. These sgRNA expression cassettes were ligated by successive Golden Gate and T5 Exonuclease-dependent assemblies [24] (Figure 1A). The tandemly ligated sgRNA cassettes were then integrated by I-*Sce* I-mediated transgenesis into the genome of embryos obtained from crossing the Tg(*zpc*:*zcas9*) homozygous male with the wild-type female. The resultant mosaic founder fish carrying both *zpc*:*zcas9* and transgenic sgRNAs in the germ-line were further outcrossed and their GFP-positive offspring were analyzed for double maternal mutations (Figure 1B).

### 3.2. Generation of dvl2 and dvl3a Double Mutants by Oocyte-Specific CKO

**Figure 2:**
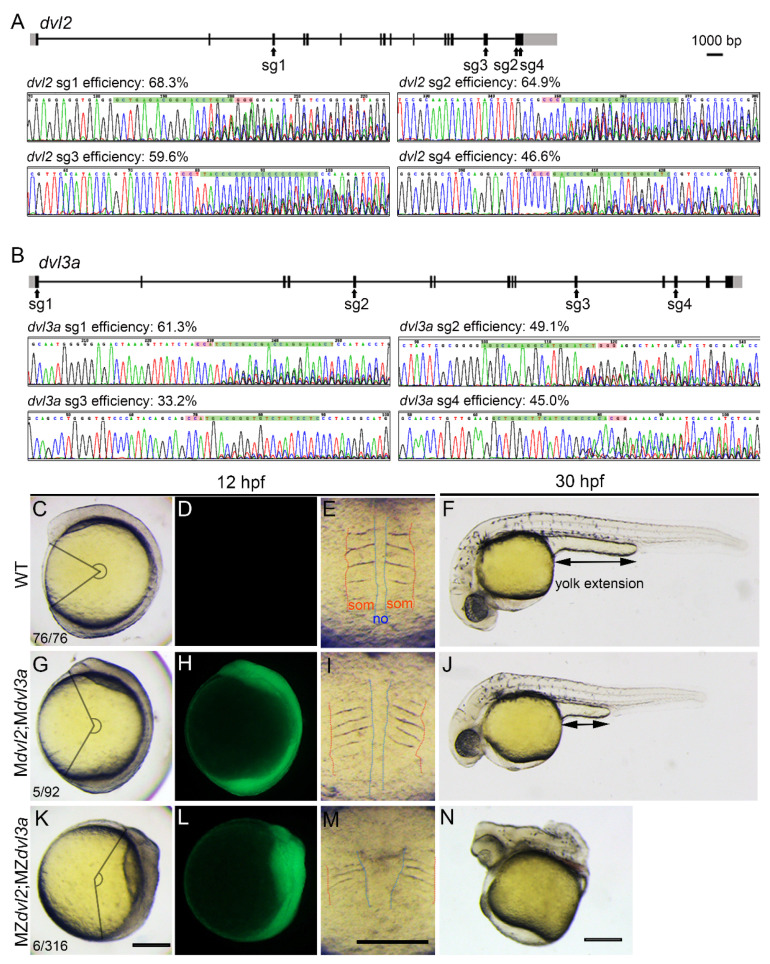
Generation of double maternal mutants of *dvl2* and *dvl3a* by oocyte-specific expression of eight Cas9 RNPs. (**A**) Gene structures of *dvl2*, with sgRNA targeting sites (sg1–4) as indicated. Black boxes represent exons, and grey boxes indicate untranslated regions. The chromatograms show indel mutations caused by each sgRNA with estimated mutation rates indicated on the top. (**B**) Positions of the four effective *dvl3a* sgRNAs and their editing efficacy after coinjection with *cas9* mRNAs. (**C**) A 12 hpf wild-type embryo. The central angle is defined by the rostral and rear end of the body axis. (**D**) No GFP expression in the wild-type (WT) embryo. (**E**) Dorsal structures of a wild-type embryo, with red lines showing the outer boundary of somites (som). Blue lines depict the shape of the notochord (no). (**F**) A wild-type embryo at 30 hpf. The bi-directional arrow indicates the length of the yolk extension. (**G**,**H**) A 12 hpf M*dvl2*;M*dvl3a* embryo with apparent axis extension defect and GFP expression. (**I**) Mediolaterally wide and anteroposteriorly compacted somites of the embryo as in (**G**). (**J**) The same double maternal mutant at 30 hpf shows a significantly shortened body length and yolk extension. (**K**,**L**) A GFP-positive MZdvl2;MZdvl3a embryo with severely affected convergence and extension movements at 12 hpf; (**M**) extremely wide notochord and compacted somites of the embryo as in (**J**); (**N**) truncated posterior body and fused eyes of the MZ*dvl2*;MZ*dvl3a* embryo at 30 hpf. Lateral views with dorsal on the right and anterior up (**C**,**D**,**G**,**H**,**K**,**L**) or dorsal up and anterior to the left (**F**,**J**,**N**). Scale bars: 200 μm.

We first screened multiple effective sgRNAs to target *dvl2* or *dvl3a*. These sgRNAs were selected by CRISPRscan online software based on scores designating their potential activity and off-target effects on the zebrafish genome [27]. These in silico optimized sgRNAs were then in vitro transcribed and were individually coinjected with *cas9* mRNA to evaluate their editing efficiency. We found that four *dvl2* sgRNAs and four *dvl3a* sgRNAs showed satisfactory editing efficiencies, as indicated by their mutation rates higher than 30% (Figure 2A,B). These eight sgRNAs were then tandemly ligated to create pGGDestISceIEG-8sgRNA(*dvl2*;*dvl3a*).

By introducing pGGDestISceIEG-8sgRNA(*dvl2*;*dvl3a*) into the genome of Tg(*zpc*:*zcas9*) via I-*Sce*-I-mediated transgenesis, we obtained eight F0 founder females that can produce GFP-positive embryos. Two females with high proportions of GFP-positive offspring and which passed quality control (see *2.5* in Materials and Methods) were selected for further analysis. Phenotypic analysis indicated that 5.4% of GFP-positive embryos (*n* = 92) from these two F0 females exhibited shortened body axes and broader dorsal structures at the four-somite stage (Figure 2C–E,G–I). Compared to wild-type siblings, these defective embryos showed a significantly reduced central angle of the body axis (Figure 2C,G). They also displayed a thicker notochord and broader somite structures (Figure 2E,I). At 30 hpf, the defective embryos showed an overall shrunken body length and shortened yolk extension (Figure 2F,J). These defective phenotypes are reminiscent of M*dvl2*;M*dvl3a* as previously reported [10], while embryos with only eight sgRNAs expression but without Cas9 did not show any developmental defect (not shown). We further crossed the GFP-positive female founders with *dvl2*^+/−^;*dvl3a*^+/−^ mutant males and obtained embryos with extremely severe axis extension defects (Figure 2K–N). Those defective embryos displayed extremely short body axes (Figure 2K), significantly broad notochords, and compacted somites at the four-somite stage (Figure 2M). At 30 hpf, these embryos developed cyclopia and truncation of posterior tissues (Figure 2N), which was highly similar to the phenotype of MZ*dvl2*;MZ*dvl3a* generated by the mosaic approach [10].

### 3.3. Genotyping the Double Maternal Mutants for dvl2 and dvl3a Genes

**Figure 3:**
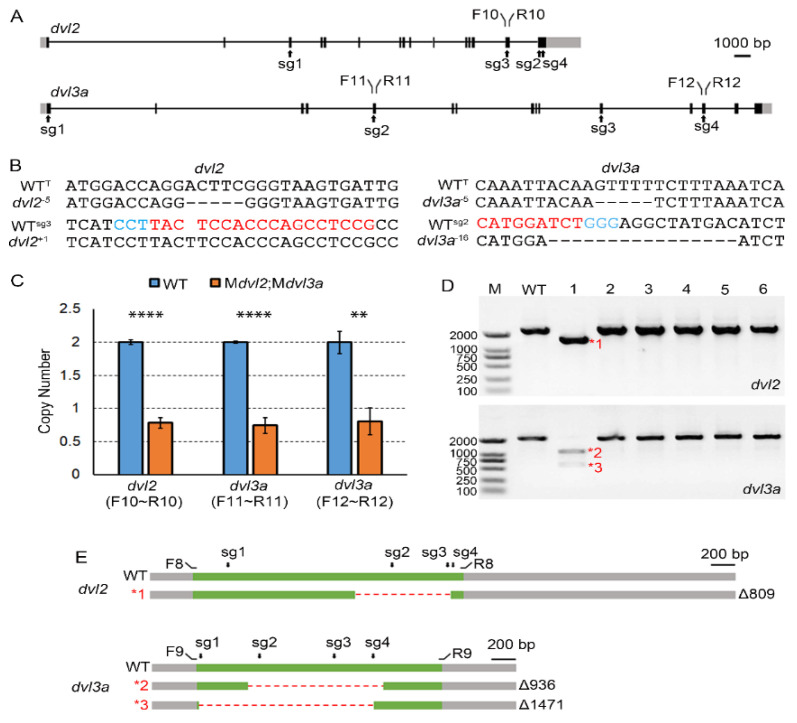
Genotyping double maternal mutants. (**A**) Gene structures of *dvl2* and *dvl3a*, with primers and sgRNA targeting sites (sg1-4) as indicated. F10, R10, F11, R11, F12, and R12 are primers used in the qPCR analysis. Black boxes represent exons, and grey boxes indicate untranslated regions. (**B**) Indels identified from a 30 hpf MZ*dvl2*;MZ*dvl3a* embryo at *dvl2* and *dvl3a* loci. WT^T^ designates the wild-type sequence near the mutation decended from male *dvl2*^+/−^;*dvl3a*^+/−^ mutant. (**C**) Ploidy determination of three genomic regions at *dvl2* and *dvl3a* loci by qPCR. An amplicon from the *dmd* gene serves as a diploid reference (**** *p* < 0.0001; ** *p* < 0.01, Student’s *t*-test). (**D**) Representative gel analysis after amplifying the coding regions of *dvl2* and *dvl3a* by RT-PCR. Two-cell stage embryos with GFP expression were individually lysed for extraction of total RNAs and RT-PCR analysis. Red asterisks and numbers designate truncated transcripts subjected to sequencing. (**E**) Mutant transcripts with large deletions observed in an M*dvl2*;M*dvl3a* mutant as shown in (**D**). Primers used to amplify the coding regions and *dvl2* and *dvl3a* sgRNAs targeting regions are marked on the wild-type cDNA. Green bars indicate coding regions, whereas grey represents untranslated regions.

To genotype the MZ*dvl2*;MZ*dvl3a*-like embryo at 30 hpf, we extracted its genomic DNA to identify mutations in *dvl2* and *dvl3a* loci by PCR amplification of sgRNA targeting sites (Figure 3A). Sequencing analysis confirmed a new +1 bp insertion at *dvl2* sgRNA3 target sequence and a new –16 bp deletion at *dvl3a* sgRNA2 site, as well as original mutations transmitted from the male *dvl2*^+/−^;*dvl3a*^+/−^ mutant (Figure 3B). We also examined the sequences of all sgRNA target sites of an M*dvl2*;M*dvl3a*-like embryo. Intriguingly, all these amplicons were wild-type as determined by sequencing. This unusual phenomenon reminded us of the deletion-prone tendency in creating *nanog* and *ctnnb2* maternal mutants [18]. Since these defective embryos were generated after crossing a GFP-positive F0 female with a wild-type male, some of the eight sgRNA targets must be in a heterozygous mutant status. If the mutant allele from the mother had large deletions of DNA fragments encompassing the *dvl2* and *dvl3a* sgRNA targets, then the PCR could only amplify the wild-type allele from the male. To test this possibility, we examined the ploidy of the two loci in the genome of the defective embryo by qPCR using an amplicon in the *dmd* gene as a diploid reference. The result showed that one position of *dvl2* and two positions of *dvl3a* tested in the defective embryo were indeed haploid (Figure 3A,C), suggesting large deletions did cover the sgRNA targeting sites of both genes. 

To further confirm the veracity of the double maternal mutant with large deletions, we individually extracted total RNAs from two-cell stage embryos with GFP expression and performed RT-PCR analysis to examine the *dvl2* and *dvl3a* coding regions from maternal transcripts. As an example of large deletions, one embryo (#1) showed truncated transcripts for both genes (Figure 3D). The truncated *dvl2* mRNA had an 809 bp deletion in the cDNA sequence (Figure 3E; *1), while *dvl3a* transcripts had two major mutant variants. The two *dvl3a* bands with lower molecular weight corresponded to mutant mRNAs with 936 bp and 1471 bp deletions, respectively (Figure 3E; *2 and *3). These data thus demonstrate a rapid generation of double maternal mutants using this oocyte-specific conditional knockout approach. They also further supported the conclusion that *dvl2* and *dvl3a* are not involved in maternal Wnt activation, which determines the formation of the dorsal organizer. In fact, the maternal and zygotic products of these two genes play redundant roles in zygotic Wnt/β-catenin and Wnt/PCP signaling that control anteroposterior axis formation and morphogenetic movements in vertebrate embryos [10].

## 4. Discussion

Functional redundancy provided by paralogs is a frequent option to ensure the robustness of embryonic development [20,28,29]. CRISPR/Cas13d was reported to induce efficient mRNA knockdown in zebrafish embryos [30], and thus showed its potential in generating multiplex maternal gene knockdowns. However, like all other knockdown strategies, Cas13d-mediated gene knockdown cannot inactivate maternally inherited proteins. It is thus necessary to develop a simple method for the generation of double maternal mutants. In zebrafish, a conditional knockout system against multiple genes is particularly beneficial because many zebrafish genes have an extra copy due to a teleost-specific whole-genome duplication event during evolution [31,32,33]. In this study, we tested the feasibility of the oocyte-specific CKO approach in generating double maternal mutants. As a proof-of-principle, we successfully obtained M*dvl2*;M*dvl3a* and MZ*dvl2*;MZ*dvl3a* in just one fish generation. Compared to the previously described mosaic approach, this CKO strategy saves time by about 80%. Most importantly, because *dvl2* and *dvl3a* double zygotic mutants are not viable, our work also presented the first evidence that this oocyte-specific CKO method can solve the difficulty caused by zygotic lethal phenotypes while generating maternal mutant offspring. 

The efficiency of this CKO method in generating double maternal mutants still has room for improvement. In this work, we observed 5.4% of embryos were M*dvl2*;M*dvl3a* among GFP-positive sgRNA expressing embryos. This is predictable, because we obtained an average of 25–38% maternal mutants for a single gene using this system [18]. So theoretically, the ratio for double maternal mutant should be below 10% when calculating the square of the mutant proportions for single genes. Hence, generating the double maternal mutant has already reached the limit of the current oocyte-specific CKO system.

Further work is thus urgently required to increase the performance of this system. Injection of sufficient amounts of Cas9 protein and four sgRNAs targeting a single gene of interest in the embryos can disrupt almost all wild-type alleles and produce phenotypes mimicking the homozygous zygotic mutants [26,34]. Thus, the moderate efficiency of CKO systems is likely to be the consequence of the suboptimal expression of the Cas9 protein or sgRNAs in the transgenic lines. Indeed, the expression level of maternally inherited Cas9 RNPs was shown to be insufficient in causing mutations of the wild-type allele from sperm after fertilization [18], highlighting the necessity of increasing the expression levels of both the Cas9 protein and sgRNAs in this CKO system.

There are several options to improve the performance of the oocyte-specific CKO system. First, Cas9 expression in the transgenic lines can be improved transcriptionally or post-transcriptionally. The transcription of *cas9* can be increased via either gal4-UAS or the Suntag system [35,36]. On the other hand, the *cas9* mRNA sequence may be modified to enhance its stability or its translation. Alternatively, sgRNA can be tandemly ligated and transcribed via strong conventional promoters, followed by processing via Csy4, ribozyme or tRNA systems to create mature sgRNAs [37,38,39]. Lastly, the dependence of I-*Sce*-I-mediated sgRNA transgenesis represents another inconvenience of this strategy, because the germ-line transmission efficiency of I-*Sce*-I-mediated transgenesis is less stable compared with that of Tol2. Hence, generating a *zpc*:*cas9* knockin line should help switch to the highly efficient Tol2 system for sgRNA transgenesis. This improvement will further lower the technical requirement in generating sgRNA expressing mosaic F0 fish.

## 5. Conclusions

The current oocyte-specific CKO strategy is helpful in generating double maternal mutants in a time-saving fashion. Improvement of this oocyte-specific CKO system will facilitate functional studies for paralogous maternal genes in zebrafish. 

## Data Availability

All data needed to evaluate the conclusions in the paper are included in the paper and/or the Supplementary Materials. The plasmid system and fish lines used in this study are available from the corresponding author upon reasonable request.

## Supplementary Materials

The following are available online at https://www.mdpi.com/article/10.3390/biology10080777/s1, Table S1: Primers used in this study.
