# Supplementary material for: A Time-Saving Strategy to Generate Double Maternal Mutants by an Oocyte-Specific Conditional Knockout System in Zebrafish
Source: Biology (Basel). 2021 Aug 16;10(8):777. doi: 10.3390/biology10080777 (PMC8389640; doi:10.3390/biology10080777)
Supplement: Supplementary file 1 [file biology-10-00777-s001.zip › biology-1291924-supplementary.pdf]

Table S1 Primers used in this study				
**Usage**	**Primer nanme**		**Sequence 5'-3'**	**note**
Construction of sgRNA expression plasmids	dvl2 sg1 fw		ttcgGCTGAGACGGGACCTCTAC	
	dvl2 sg1 rv		aaacGTAGAGGTCCCGTCTCAGC	
	dvl2 sg2 fw		ttcgGTGGGGGTAAGCCAGGGTG	
	dvl2 sg2 rv		aaacCACCCTGGCTTACCCCCAC	
	dvl2 sg3 fw		ttcgGCGGAGGCTGGGTGGAGTA	
	dvl2 sg3 rv		aaacTACTCCACCCAGCCTCCGC	
	dvl2 sg4 fw		ttcgGGAGCCCAGGTCTCGGGTC	
	dvl2 sg4 rv		aaacGACCCGAGACCTGGGCTCC	
	dvl3a sg1 fw		ttcgGGTTTCCTGGTCGTCGAGA	
	dvl3a sg1 rv		aaacTCTCGACGACCAGGAAACC	
	dvl3a sg2 fw		ttcgGAGGCAGAGGCATGGATCT	
	dvl3a sg2 rv		aaacAGATCCATGCCTCTGCCTC	
	dvl3a sg3 fw		ttcgGGAGGATAGACACCCGTCA	
	dvl3a sg3 rv		aaacTGACGGGTGTCTATCCTCC	
	dvl3a sg4 fw		ttcgGCTGGCTTCATCCGCCACA	
	dvl3a sg4 rv		aaacTGTGGCGGATGAAGCCAGC	
sgRNA template primer for in vitro transcription	dvl2_Target_1		TAATACGACTCACTATAGGCTGAGACGGGACCTCTACGTTTTAGAGCTAGAA	
	dvl2_Target_2		TAATACGACTCACTATAGGTGGGGGTAAGCCAGGGTGGTTTTAGAGCTAGAA	
	dvl2_Target_3		TAATACGACTCACTATAGGCGGAGGCTGGGTGGAGTAGTTTTAGAGCTAGAA	
	dvl2_Target_4		TAATACGACTCACTATAGGGAGCCCAGGTCTCGGGTCGTTTTAGAGCTAGAA	
	dvl3a_Target_1		TAATACGACTCACTATAGGGTTTCCTGGTCGTCGAGAGTTTTAGAGCTAGAA	
	dvl3a_Target_2		TAATACGACTCACTATAGGAGGCAGAGGCATGGATCTGTTTTAGAGCTAGAA	
	dvl3a_Target_3		TAATACGACTCACTATAGGGAGGATAGACACCCGTCAGTTTTAGAGCTAGAA	
	dvl3a_Target_4		TAATACGACTCACTATAGGCTGGCTTCATCCGCCACAGTTTTAGAGCTAGAA	
Genomic DNA fragment amplification	dvl2 1 fw1		GACCTGAAGCAATGCGCGATCA	amplify sg1 site
	dvl2 1 rv1		CAATACTCACAAATGCAGCTTC	
	dvl2 2 fw1	F10	CTCTCAATGACAATGATGGGTC	amplify sg3 site
	dvl2 2 rv1	R10	GTCAGTGTAGCTCCTCACCTTC	
	dvl2 3 fw1		GATGTGATGTAATGAAGGATGTA	amplify sg2 & sg4 site
	dvl2 3 rv2		TGAAGTCTGTGCTATGCAAGACT	
	dvl2 3 rv1		ACTACATCTCTGTCCGTCGCAG	
	dvl3a 1 fw		GAGTACGGATTAATAATCACCT	amplify sg1 site
	dvl3a 1 rv		TCTGGAAGATCATGGCAGTCGAT	
	dvl3a 2 fw	F11	CAAGCCACAGTGGCTAGTGAGC	amplify sg2 site
	dvl3a 2 rv	R11	CTGTGAGTCTCACCGGCTGGTG	
	dvl3a 3 fw		ACTCGCCTGTTCTGCTCAGCTT	amplify sg3 site
	dvl3a 3 rv		AGCAGAAGTCTTACTCTCGGTC	
	dvl3a 4 fw	F12	GCAGGTTCGGATGTGGTGGACT	amplify sg4 site
	dvl3a 4 rv	R12	CAGAGAATCTCATAGGAGGCTA	
	dmd fw		AGACGTCTTCTCCTCGTT	

	dmd rv		TTGCCTTTTAATATGCATTTGTTATTTTG	
CDS amplification in RT-PCR	dvl2 cds fw	F8	ATGGCGGAGACCAAGATAATTTATC	
	dvl2 cds rv	R8	CATCACATCCACAAAAAACTCAC	
	dvl3a cds fw	F9	ATGGGGGAGACTAAAGTTATC	
	dvl3a cds rv	R9	CATGATGTCGACGAAGAAC	
